# Construction and validation of the prognostic nomogram model for patients with diffuse-type gastric cancer based on the SEER database

**DOI:** 10.1007/s12672-024-01180-0

**Published:** 2024-07-24

**Authors:** Ting Huang, ChuiPing Chan, Heran Zhou, Keke Hu, Lu Wang, Zhifeng Ye

**Affiliations:** 1 The Third School of Clinical Medicine (School of Rehabilitation Medicine), Zhejiang Chinese Medical University, Hangzhou, China; 2 Hangzhou TCM Hospital of Zhejiang Chinese Medical University (Hangzhou Hospital of Traditional Chinese Medicine), Hangzhou, China

**Keywords:** Diffuse gastric cancer, SEER, Prognosis, Nomogram

## Abstract

**Objective:**

The prognostic factors of diffuse GC patients were screened the prognostic nomogram was constructed, and the prediction accuracy was verified.

**Methods:**

From 2006 to 2018, there were 2877 individuals pathologically diagnosed with diffuse gastric cancer; the clinicopathological features of these patients were obtained from the SEER database & randomly divided into a training cohort (1439) & validation cohort (1438).To create prognostic nomograms & choose independent prognostic indicators to predict the overall survival (OS) of 1, 3, & 5 years, log-rank & multivariate COX analysis were utilized & discrimination ability of nomogram prediction using consistency index and calibration curve.

**Results:**

Age, T, N, M, TNM, surgical status, chemotherapy status, & all seven markers were independent predictors of OS (P < 0.05), & a nomogram of OS at 1, 3, & 5 years was created using these independent predictors. The nomogram's c-index was 0.750 (95% CI 0.734 ~ 0.766), greater than the TNM staging framework 0.658 (95%CI 0.639 ~ 0.677); the c-index was 0.753 (95% CI 0.737 ~ 0.769) as well as superior to the TNM staging mechanism 0.679 (95% CI 0.503–0.697). According to the calibration curve, the projected survival rate using the nomogram & the actual survival rate are in good agreement.

**Conclusions:**

Prognostic nomograms are useful tools for physicians to assess every individual's individualised prognosis & create treatment strategies for those with diffuse gastric cancer. They can reliably predict the prognosis for individuals with diffuse gastrointestinal carcinoma.

## Introduction

Diffuse gastric cancer, which primarily affects young & middle-aged women in China, is becoming more common every year [1–5]. A retrospective study of 2379 gastric cancer patients found that 78.6% of local gastric cancer patients (18–45 years old) had diffuse gastric cancer [6]. A larger percentage of advanced stomach cancers are diffuse. Diffuse gastric cancer progresses rapidly, and lymph node or distant metastasis can occur in the early stage. Comprehensive treatment based on surgical treatment is the main way at present. The operation is mainly radical total gastrectomy [7, 8]. If adjacent organs are invaded or distant metastasis occurs, combined organ resection should be considered. The main reason for the failure of surgical treatment is local recurrence [9]. The recurrent cancer focus is mainly located in the cancer bed, anastomotic stump and regional lymph nodes, accounting for about 65% of all patients with recurrence and metastasis in total, so the radical surgery rate is low and the prognosis is poor [10]. Diffuse GC is a subtype of GC with an aggressive nature and poor prognosis, and it is urgent to screen adverse prognostic factors for diffuse GC to guide individualized treatment options [11, 12].

In clinical practice, in order to facilitate standardized treatment of tumors, tumor staging has emerged. Tumor staging is based on the pathological diagnosis results as the evaluation basis, not just on the intuitive size of the tumor. There are many types of tumor staging, and the current common standard is TNM staging. The TNM staging system is currently the most commonly used tumor staging system internationally, and it is also the standard method for staging malignant tumors in clinical practice. The TNM staging method was first proposed by Pierre Denoix, a Frenchman, between 1943 and 1952. Subsequently, the American Joint Commission on Cancer (AJCC) and the International Union Against Cancer (UICC) began establishing international staging standards.

Prognostic nomograms are being used often to forecast the prognosis of cancer individuals and offer a greater degree of predictability than conventional clinical staging [13–15]. Accurately assessing the risk of disease occurrence and patient prognosis can assist clinical doctors in early intervention and treatment of the disease. In recent years, column charts have received increasing attention and application as a tool for assessing disease risk and prognosis in medical research and clinical practice. A nomogram, also known as a nomogram, is a method of constructing a multiple factor regression model. Based on the degree of influence of each variable on the outcome event in the model, each value level of each influencing factor is assigned a score, and then the total score is obtained by adding up the scores. The predicted probability of the individual's outcome event is calculated by converting the function between the total score and the probability of the outcome event. Finally, the predicted model is presented in a graphical form. After transforming complex regression equations into visual graphs using column charts, clinical doctors can easily calculate the probability of disease occurrence and judge the prognosis of patients based on the graphs. At present, various column charts for different tumors have been widely used, and their evaluation of tumor prognosis is even comparable to traditional TNM staging systems. In this work, we looked at the data from individuals who had diffuse gastric cancer in the National Cancer Research SEER database (surveillance, epidemiology, & end outcomes, SEER), picked prognostic markers, & created prognostic nomograms.

## Materials and methods

### Data sources

Using SEER * Stat software (version 8.3.9), clinical and pathological data of diffuse gastric cancer patients diagnosed from 2006 to 2018 were collected. The data record includes the patient's registration number, personal information, location of the primary lesion, tumor size, tumor code, treatment plan, cause of death, and other information. Any information shall be verified and jointly entered by two individuals.

### Object of study

The following 14 variables were extracted from the patient's records: age, sex, T (scope of primary tumour invasion), N (regional lymph node), M (distant metastasis), primary lesion, TNM (stage), pathological grade, radiotherapy status, surgical status, chemotherapy status, marital status, survival time, survival status, & pathological type.

Criteria for Inclusion: (1) diagnosis of gastric adenocarcinoma histologically; (2) tumor diagnosis from 2006 to 2018; (3) gastric cancer was the first primary and the only malignant tumor; (4) a definite survival time; (5) pathological type was included in 8145 / 3 diffuse gastric cancer according to the ICD-0–3 standard for International Classification of Oncology-Diseases; (6) marital status was divided into: married, unmarried, divorced, separated, or widowed.Exclusion criteria: (1) survival or follow-up time of less than 1 month; (2) unclear clinical stage; (3) primary surgery, unclear radiotherapy and chemotherapy status; (4) unclear marital status. The study comprised 2877 individuals who had diffuse gastric cancer, who were split into two cohorts: a training group (1439) & a validation group (1438).

### The best cut-off value

The ages of 2877 diffuse gastric cancer patients were analyzed using x-tile software. Generate X-tileplot and histogram, divide them into 3 groups, and obtain the corresponding Kaplan Meier curves. The corresponding truncation values can be obtained through the exported table and histogram.

### Statistical treatment

The 2 tests in SPSS (version 22.6) software were used to identify the differences in clinicopathological traits among the validation as well as training cohorts. It included univariate Log-rank analysis & multivariate Cox analysis to assess the factors affecting OS & create survival curves of Kaplan–Meier. The R language (version 4.1.0) builds the OS prognostic nomogram, calculates the c index (c-index) and draws the calibration curve, and the Bootstrap method performs internal (training queue) and external (validation queue) validation. Validation based on model development queue data to verify the repeatability of the model development process and prevent overestimation of model performance due to overfitting. For all steps in the entire modeling process, including variable transformation, variable screening, model selection, and imputation of missing data values, using data segmentation or resampling methods, the model development queue is randomly divided into two parts: the training set and the validation set, with a ratio of 1:1. P < 0.05 indicates a statistical significance.

## Results

### The optimal cutoff value for age

**Fig. 1:**
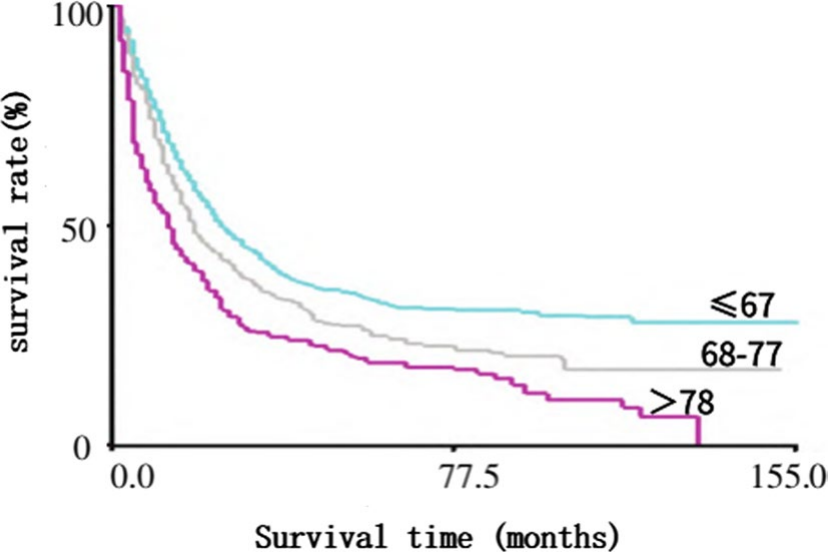
Survival analysis of optimal cutoff for age of diagnosis in diffuse gastric cancer (n = 2877). The green, gray and red lines represent the survival of patients aged 67,68–77 and > 77 years, correspondingly, & the variance was noteworthy (P < 0.05)

The optimal cutoff for age (training cohort) was 67 and 77 years, divided into three groups (67,68–77, > 77). See Fig. 1.

### Clinical Characteristics & prognosis in individuals with widespread cancer of the stomach

All of the individuals receiving treatment were 62 years old on average and mainly married people were 67 years. Most of the first diagnosed patients had low/undifferentiated pathological grades and no distant metastasis. Most patients received surgery for the primary focus, a few received radiotherapy, and more than half received chemotherapy. Clinical traits across the training group & validation group did not change significantly (P > 0.05). Look at Table 1.

**Table 1:** Clinicopathological topographies of patients with diffuse-type gastric cancer [n (%)]

variable	Training cohort (n = 1439)	Internal validation queue (n = 1438)	χ^2^	P
Age (year)				
≤ 67	933(64.8)	923(64.2)	0.146	0.93
68–77	293(20.4)	300(20.9)		
> 77	213(14.8)	215(15.0)		
Sex				
Male	746(51.8)	763(53.1)	0.428	0.513
Female	693(48.2)	675(46.9)		
T				
T1	277(19.2)	298(20.7)	1.048	0.79
T2	446(31.0)	435(30.3)		
T3	426(29.6)	415(28.9)		
T4	290(20.2)	290(20.2)		
N				
N0	511(35.5)	533(37.1)	1.672	0.643
N1	450(31.3)	419(29.1)		
N2	293(20.4)	295(20.5)		
N3	185(12.9)	191(13.3)		
M				
M0	1132(78.7)	1122(78.0)	0.174	0.677
M1	307(21.3)	316(22.0)		
TNM				
I	338(23.5)	369(25.7)	5.626	0.131
II	260(18.1)	219(15.2)		
III	411(28.6)	398(27.7)		
IV	430(29.9)	452(31.4)		
Pathological grading				
High / medium differentiation	37(2.6)	28(1.9)	1.269	0.26
Low/undifferentiated	1402(97.4)	1410(98.1)		
Surgical status of the primary focus				
No	286(19.9)	302(21.0)	0.561	0.454
Yes	1153(80.1)	1136(79.0)		
Radiotherapy status				
No	1019(70.8)	1038(72.2)	0.688	0.709
Preoperation radiotherapy	37(2.6)	34(2.4)		
Postoperation radiotherapy	383(26.6)	366(25.5)		
Chemotherapy status				
No	691(48.0)	730(50.8)	4.014	0.26
Pre-operative chemotherapy	135(9.4)	136(9.5)		
Postoperative chemotherapy	521(36.2)	471(32.8)		
Preoperative and postoperative chemotherapy	92(6.4)	101(7.0)		
Marital status				
Unmarried	218(15.1)	260(18.1)	7.422	0.115
Married	904(62.8)	879(61.1)		
Divorce	148(10.3)	126(8.8)		
Separation	22(1.5)	31(2.2)		
Bereft of one's spouse	147(10.2)	142(9.9)		

During the follow-up period of 1 to 155 months, At ages 1, 3, & 5, the overall survival rates were 64.3% (1850 / 2877), 36.4% (1047 / 2877), 28.8% (829 / 2877), respectively, Stage I, 1, 3, & 5-year TNM stage overall survival rates were 81.9%, 66.3% and 58.8%; The yearly overall survival rates for periods 1, 3, and 5 were 81.0%, 50.7%, & 39.8%, correspondingly; for periods 1, 3, & 5 years, the rates were 63.5%, 31.6%, & 19.1%, correspondingly; & for stages 1, 3, & 5 years, the rates were 43.3%, 9.5%, & 5.4%, correspondingly.

### Prognostic factors in diffuse-type gastric cancer patients

The outcomes of univariate analysis show, age, T, N, M, TNM stage, pathological grade, surgical status, radiotherapy status, chemotherapy status, and marital status were all possible factors influencing the rate of survival (P < 0.05). Additional multivariate analyses revealed that age, T, N, M, TNM, surgical status, & Each of these independent variables affected the survival rate in their unique ways (P < 0.05). Among them, advanced age, number of regional lymph node metastases, distant metastasis, late clinical-stage, low/undifferentiated, no primary surgery, no postoperative radiotherapy, no chemotherapy, & widowed were all survival risk factors (P < 0.05). See Table 2.

**Table 2:** The individual survival rate in the training category: a multivariate & univariate analysis

variable	Univariate analysis (P-value)	multiplicity HR(95% CI)	P
Age (year)			
≤ 67	consult	consult	< 0.001
68–77	< 0.001	1.561(1.317 ~ 1.852)	< 0.001
> 77	< 0.001	1.817(1.483 ~ 2.226)	< 0.001
Sex			
Male	consult	consult	
Female	0.537	Not carried out	0.002
T			
T1	consult	consult	
T2	< 0.001	1.395(1.097 ~ 1.774)	0.007
T3	< 0.001	1.479(1.121 ~ 1.952)	0.006
T4	< 0.001	1.735(1.311 ~ 2.296)	< 0.001
N			
N0	consult	consult	
N1	0.002	0.989(0.807 ~ 1.213)	0.915
N2	< 0.001	1.231(0.962 ~ 1.574)	0.098
N3	< 0.001	1.369(1.019 ~ 1.840)	0.037
M			
M0	consult	consult	0.005
M1	< 0.001	1.481(1.127 ~ 1.946)	< 0.001
TNM			
I	consult	consult	
II	0.003	1.555(1.149 ~ 2.103)	0.004
III	< 0.001	2.316(1.661 ~ 3.230)	< 0.001
IV	< 0.001	2.691(1.831 ~ 3.955)	< 0.001
Pathological grading			
High / medium differentiation	consult	consult	
Low/undifferentiated	0.017	1.542(0.938 ~ 2.535)	0.088
Surgical status of the primary focus			
No	consult	consult	< 0.001
Yes	< 0.001	0.434(0.350 ~ 0.537)	0.05
Radiotherapy status			
No	consult	consult	0.015
preoperation radiotherapy	0.937	1.712(1.112 ~ 2.635)	0.977
postoperation radiotherapy	< 0.001	1.003(0.822 ~ 1.223)	< 0.001
Chemotherapy status			
No	consult	consult	0.028
Pre-operative chemotherapy	< 0.001	0.728(0.548 ~ 0.967)	< 0.001
Postoperative chemotherapy	< 0.001	0.581(0.476 ~ 0.710)	< 0.001
Preoperative and postoperative chemotherapy	< 0.001	0.444(0.314 ~ 0.627)	0.555
Marital status			
Unmarried	consult	consult	
Married	0.607	1.008(0.828 ~ 1.227)	0.937
Divorce	0.86	1.132(0.865 ~ 1.482)	0.367
Separation	0.281	1.302(0.727 ~ 2.330)	0.374
Bereft of one's spouse	0.007	1.15(0.876 ~ 1.511)	0.314

### Construction & validation of a nomogram

#### Construct a prognostic nomogram

**Fig. 2:**
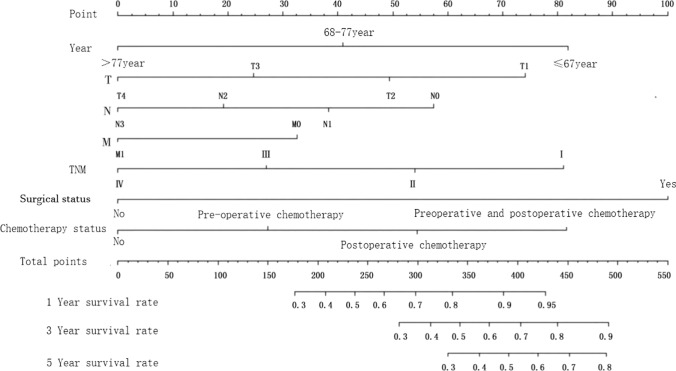
1,3, & 5-year nomograms constructed based on independent factors influencing survival of diffuse gastric cancer patients

The predictive nomogram was developed & illustrated using age, T, N, M, TNM, surgical status, and chemotherapy status, and obtained at 1,3 and 5 years. After the sum of the scores, the survival rate of 1,3 and 5 years corresponding to the total score scale below the nomogram was compared. See Fig. 2.

### Test and verify

**Fig. 3:**
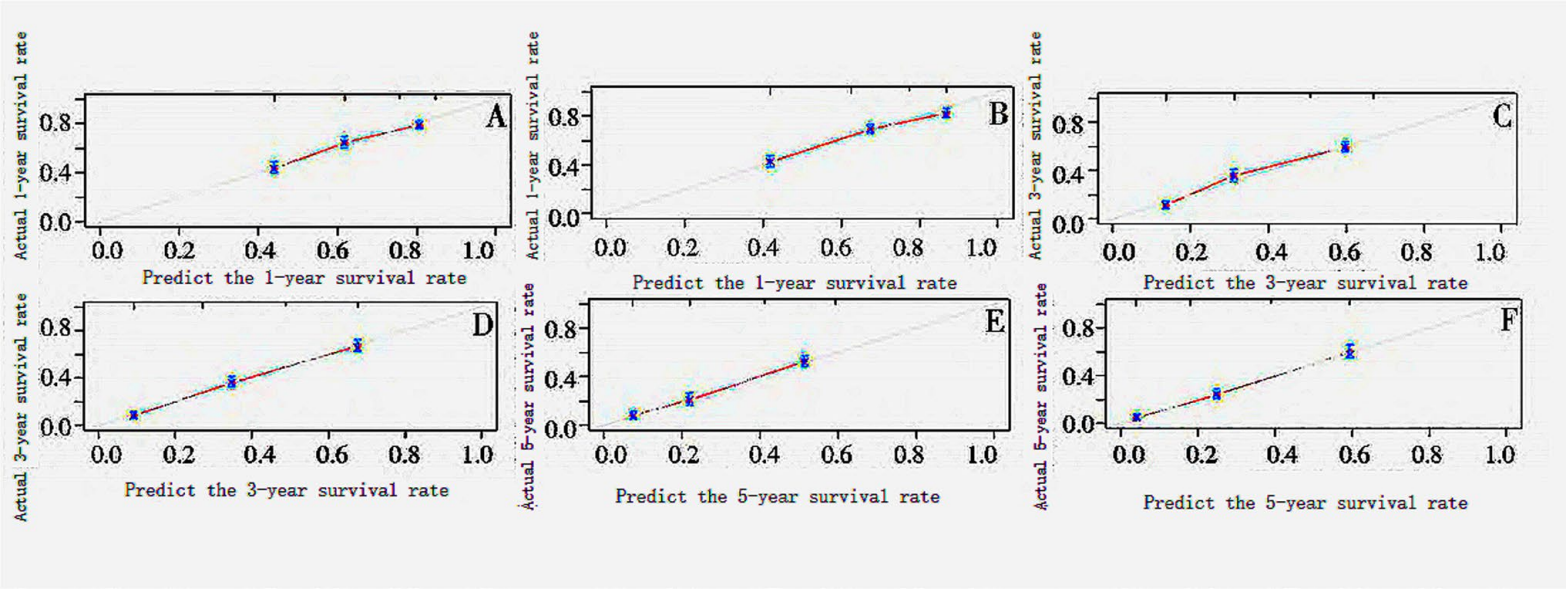
Calibration curve for survival in the prognosis nomogram 1,3,5 of diffuse gastric cancer patients. **A**, **C**, **E** training cohorts; **B**, **D**, **F** validation cohorts

The internal & external validation of the prognostic nomogram model was carried out following its design using the Bootstrap technique. The c-index of the nomogram in the training cohort was 0.750 (95% CI 0.734 to 0.766), 0.658 (95% CI 0.639 ~ 0.677); The c-index of the validation cohort nomogram was 0.753 (95% CI 0.737 to 0.769), It was 0.679 (95% CI 0.503–0.697) points higher than the TNM staging method. In terms of the projected & actual observed values, the calibration findings indicated a fair degree of agreement among the training cohort & the validation group. Examine Fig. 3.

## Discussion

One kind of gastric cancer is diffuse gastric carcinoma. Lauren typing, it was shown that cancer cells in the gastric cancer stem cell enrichment of gastric proliferative region are derived from the stomach mucosa, high degree of malignancy, more for low differentiated carcinoma, undifferentiated carcinoma or signet ring cell carcinoma, the disease progresses rapidly, more in the early stage of peritoneal metastasis, lymphatic system and distant organ metastasis [16–20]. Some studies have shown that diffuse gastric malignancy is greatly prejudiced by hereditary factors, and some patients with diffuse gastric cancer show familial aggregation and heredity, which is called hereditary diffuse gastric cancer [21–25]. Biological behavior shows diffuse growth, forming the "leather sac stomach", low surgical resection rate, and extremely poor prognosis [26–30]. To more accurately forecast the survival rate of individuals who have diffuse gastric cancer, our study incorporates independent prognostic markers in these patients & creates a prognostic nomogram model [31–33].

Age, T, N, M, TNM, stage, seven features of surgical status, and chemotherapy status were all independent prognostic factors for diffuse-type gastric cancer. Individuals with diffuse cancer of the stomach had an ideal cut-off age between 67 & 77 years old, and the prognosis of patients over 67 years was poor [34, 35]. This may be related to multiple factors such as more underlying diseases, decreased treatment tolerance and decreased treatment willingness in elderly patients. As a crucial determinant to assess the prognosis of tumour individuals, the clinical TNM stage is used for the prognosis evaluation of almost all solid tumors. The T, N, M and TNM stage in this study are closely related to the patient prognosis, Moreover, the latter stage frequently indicates a bad prognosis, which is in line with earlier findings [36]. At present, treatment methods for gastric cancer mainly include surgical resection, systemic chemotherapy, radiotherapy and others [37]. For patients with early-stage or locally advanced gastric cancer, surgery remains the cornerstone of care [38]. In addition to being the primary therapy for postoperative & advanced stomach cancer, chemotherapy is also crucial for preoperative care. In this study, individuals with surgery or chemotherapy often had a longer survival period, suggesting that surgery and chemotherapy are important treatments for patients with diffuse gastric cancer. Moreover, radiotherapy, as one of the treatments for gastric cancer, can improve the surgical resection rate and survival time of patients. In this study, postoperative radiotherapy improved patient survival time, but preoperative radiotherapy did not bring survival benefits. Some studies have found that preoperative radiotherapy can reduce tumor stage and improve tumor resection rate [39], and there is no difference in the survival of patients receiving preoperative and postoperative radiotherapy compared with patients receiving postoperative radiotherapy [40–42]. Therefore, the role of radiotherapy remains to be studied.

In this study, we combined independent prognostic characteristics of patients with diffuse gastric cancer, including advanced age, number of regional lymph node metastases, distant metastasis, clinical advanced stage, low/undifferentiated, no primary surgery, no postoperative radiotherapy, no chemotherapy, widowhood, etc., age, T, N, M, TNM, surgical status, and chemotherapy status, and created 1-year, 3-year, and 5-year survival rate nomograms, respectively. The prognostic column chart model was internally and externally validated, and the results showed that it accurately predicted the survival rate of individual patients, indicating that the model has high predictive accuracy and is beneficial for clinical doctors to evaluate the individualized prognosis of patients and develop treatment plans. Compared to traditional TNM staging and other staging methods, the survival rate column chart provides a more accurate and personalized indication. This prediction model is superior to TNM staging because it includes other factors and is an upgraded version.

A column chart is built on the basis of multiple regression analysis, integrating multiple predictive indicators, and then using scaled line segments to draw on the same plane in a certain proportion, in order to express the interrelationships between variables in the predictive model. Its advantage lies in the ability to directly use graphics to calculate the value of a certain variable, such as the patient's indicator score or survival probability. The column charts for this study were obtained at the ages of 1, 3, and 5 based on age, T, N, M, TNM, surgical status, and chemotherapy status, respectively. These are all prognostic factors, suggesting that in a sense, for some patients with better prognosis, more medical resources should be invested in treatment to ensure treatment effectiveness. For patients with poor prognosis, such as those with higher tumor staging or older age, more attention should be paid after treatment to ensure that they can achieve better postoperative survival and quality.

This research has several restrictions. First of all, no data on Chinese individuals are included in this research, which concentrates on foreigners. Second: The Cox analysis did not exclude the correlations between the subvariables. In addition, in recent years, the intervention of immunotherapy and targeted therapy has gradually increased, and this information has not been collected in the SEER database. More studies can bring benefits to patients.

## Conclusions

The prognostic markers for patients with diffuse gastric cancer include, in brief, age, stage, surgical status, & chemotherapy status. The prognosis nomogram can achieve a more accurate prognosis and measure the survival rate of patients, which is conducive to clinicians making individualized prognosis risk assessments of patients and formulating treatment approaches.

## Data Availability

The data could be obtained by contacting the corresponding author.
